# DiI staining of sensory neurons in the entomopathogenic nematode *Steinernema hermaphroditum*

**DOI:** 10.17912/micropub.biology.000516

**Published:** 2022-02-24

**Authors:** Pranjal Garg, Chieh-Hsiang Tan, Paul W. Sternberg

**Affiliations:** 1 Division of Biology and Biological Engineering, California Institute of Technology, 1200 East California Boulevard, Pasadena, CA 91125, USA; 2 Current Address: All India Institutes of Medical Sciences, Rishikesh, Virbhadra Road, Rishikesh, Uttarakhand 249203, India

## Abstract

*Steinernema hermaphroditum* entomopathogenic nematodes (EPN) and their *Xenorhabdus griffiniae* symbiotic bacteria have recently been shown to be a genetically tractable system for the study of both parasitic and mutualistic symbiosis. In their infective juvenile (IJ) stage, EPNs search for insect hosts to invade and quickly kill them with the help of the symbiotic bacteria they contain. The mechanisms behind these behaviors have not been well characterized, including how the nematodes sense their insect hosts. In the well-studied free‑living soil nematode *Caenorhabditis elegans*, ciliated amphid neurons enable the worms to sense their environment, including chemosensation. Some of these neurons have also been shown to control the decision to develop as a stress-resistant dauer larva, analogous to the infective juveniles of EPNs, or to exit from dauer and resume larval development. In *C. elegans* and other nematodes, dye-filling with DiI is an easy and efficient method to label these neurons. We developed a protocol for DiI staining of *S. hermaphroditum* sensory neurons. Using this method, we could identify neurons positionally analogous to the *C. elegans* amphid neurons ASI, ADL, ASK, ASJ, as well as inner labial neurons IL1 and IL2. Similar to findings in other EPNs, we also found that the IJs of *S. hermaphroditum* are dye-filling resistant.

**Figure 1. Optimized DiI staining labels amphid and inner labial neuron analogs of S. hermaphroditum:**
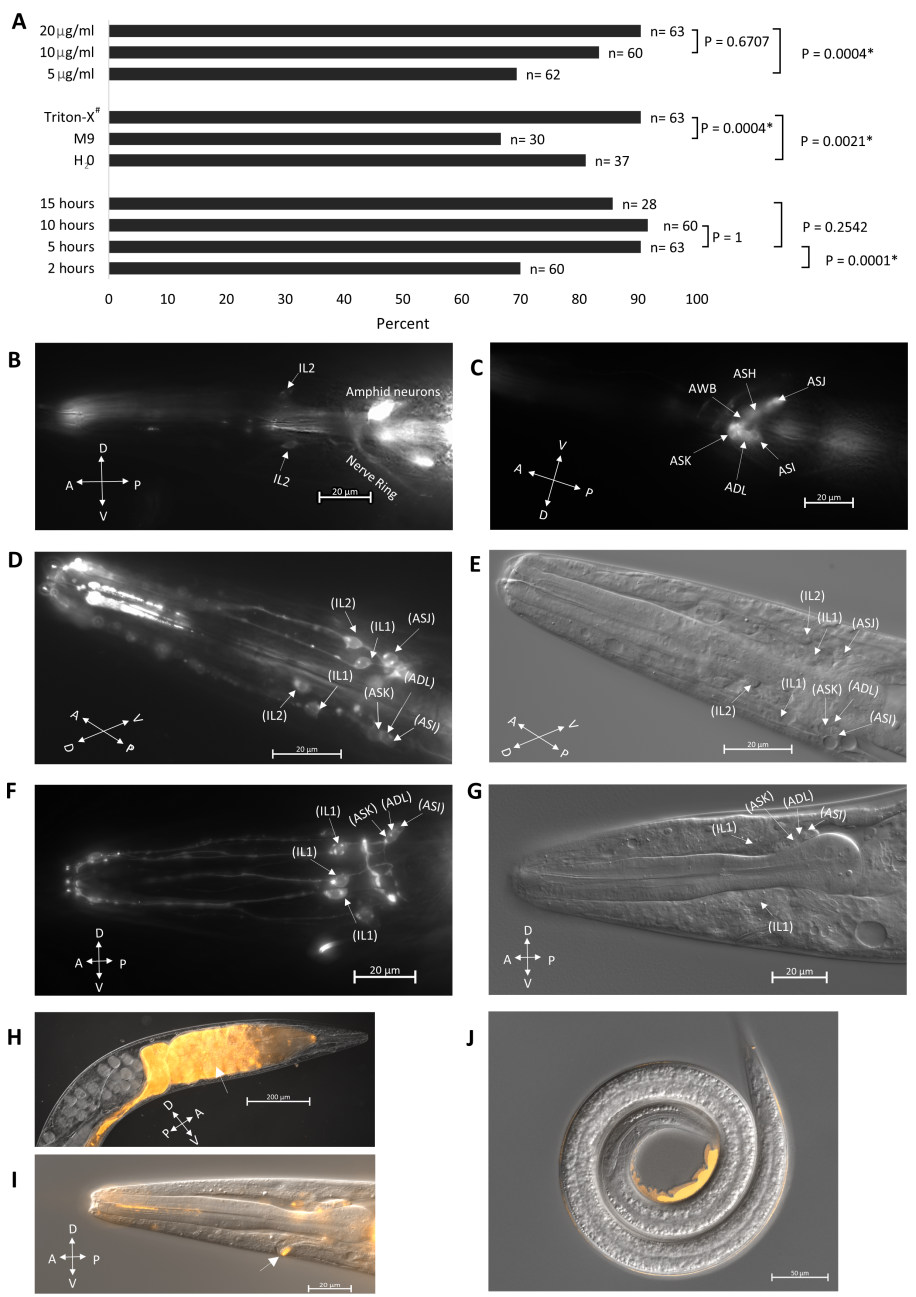
(A) Optimization of DiI head sensory neuron staining in *S. hermaphroditum*. The procedure was optimized for dye concentration (top 3 rows), buffers (middle 3 rows), and staining duration (bottom 4 rows) Living worms with at least one stained neuron and associated dendrite were scored as positive. P values are from the Fisher exact test. *: statistically significant. #: 0.5% Triton X-100 in M9. (B, C) Dye filling in the head neurons of *C. elegans*. Arrows indicate identified neurons. The anterior of the worm is to the left and in (B) dorsal is up and in (C) ventral is up. Scale bar, 20µm. (D, F) Dye filling in the head region of *S. hermaphroditum*, arrows indicate neurons identified (in parentheses) based on positional analogy to *C. elegans* counterparts. Scale bar, 20µm. (E, G) DIC (differential interference contrast) images of (D) and (F), respectively. Scale bar, 20µm. In (D, E) the anterior of the worm is to the top-left and the dorsal is bottom-left. Worm is lying in a twisted position on the agar pad. (F, G) the anterior of the worm is to the left and the dorsal is up. (H) Adult worm with dye filling (arrow) in the gut. The anterior of the worm is to the top-right and the dorsal is top-left. The dye filled gut is partly obscured by the gonad. Scale bar, 200µm. (I) DiI reliably fills the excretory pore (arrow). The anterior of the worm is to the left and dorsal is up. Scale bar, 20µm. (J) Infective juveniles (IJs) are dye-filling resistant. The staining seen at the posterior end of the animal is a shed cuticle from molting. Scale bar, 50µm. (H-J) Images were merged from DIC and DiI images. “V” Ventral, “D” Dorsal, “P” Posterior, “A” Anterior.

## Description

Entomopathogenic nematodes (EPNs) form species-specific mutualistic symbiotic relationships with their gut bacteria; on the other hand, they are parasitic to insects, which they infect and rapidly kill with the assistance of their symbiotic bacteria (Dillman and Sternberg, 2012). Studies of EPNs can therefore provide useful insight into parasitism and mutualistic symbiosis. Research in this field has been so far limited by the lack of a genetically tractable system on the host side (Cao *et al.*, 2021). *S. hermaphroditum* was first isolated in Indonesia (Griffin *et al.*, 2001); that isolate was subsequently lost, but the species was recently rediscovered in India (Bhat *et al.*, 2019). It is consistently hermaphroditic, and techniques and methods developed to study hermaphroditic *Caenorhabditis elegans* are largely applicable (Cao *et al.*, 2021). Together with its bacterial symbiote *Xenorhabdus griffiniae* it provides a genetically tractable system for the study of symbiosis.

Nematode development consists of four larval/juvenile stages, known as either L1, L2, L3, and L4 (as in *C. elegans*), or as J1, J2, J3, and J4 (as in *S. hermaphroditum*). As an alternative to the third larval stage, nematodes can become a stress-resistant form known as dauers in *C. elegans* and infective juveniles (IJs) in EPNs in response to environmental conditions including limited food. As a crucial part of their life cycle, IJs search, invade, colonize, and subsequently feed on their insect host. Chemosensation is thought to play a major role in EPNs’ host-seeking behavior, and molecules including CO_2_ have been identified as attractants for EPNs (Dillman *et al.*, 2012; Gang and Hallem, 2016; Hallem *et al.*, 2011).

The nervous system of the nematodes is highly conserved across species. For example, the tiny (~1mm) free-living nematode *C. elegans* has a neuroanatomy highly similar to that of the much larger (up to 40cm), evolutionary distant, and parasitic *Ascaris* (Schafer, 2016; Stretton *et al.*, 1992; White *et al.*, 1986). The functionality of nematode neurons are at least partially conserved. For example, ablation of the BAG neurons of both *Steinernema* and *Heterorhabditis* EPNs, identified by their conserved anatomy as corresponding to the *C. elegans* neuron responsible for sensing CO_2 _eliminated the EPNs’ CO_2_‑dependent attraction by insect hosts and the jumping response of *Steinernema carpocapsae* to CO_2 _(Hallem *et al.*, 2011). In *C. elegans*, amphid neurons are known to be important for chemosensation (Bargmann and Horvitz, 1991a), which is required for the decision-making process of entering and exiting the dauer larval state (Bargmann and Horvitz, 1991b). In *C. elegans* and other nematodes, including *Steinernema carpocapsae*, a dye-filling assay has been shown to be an efficient assay to label some of the amphid neurons (Han *et al.*, 2015; Hedgecock *et al.*, 1985; Perkins *et al.*, 1986; Srinivasan *et al.*, 2008; Tong and Burglin, 2010). In this study, we have developed a protocol for the efficient DiI (1,1′-Dioctadecyl-3,3,3′,3′-Tetramethylindocarbocyanine Perchlorate, Invitrogen) staining of *S. hermaphroditum* head sensory neurons.

We adapted the protocol described by Tong and Burglin (2010) for DiI staining, which has been utilized for the staining of the closely related nematode *S. carpocapsae* (Han *et al.*, 2015). We optimized the staining protocol by altering the concentration of the DiI dye (Fig. 1A, top), buffer solution, (Fig. 1A middle) and staining duration (Fig. 1A bottom). We found that a 20µg/ml DiI concentration with 0.5% Triton X-100 in M9 for 5 hours gave us the best results when considering both the staining effectiveness (Fig. 1A) and the health of the worm. In *C. elegans*, head sensory neurons, including amphids- ASI, ADL, ASK, AWB, ASH, ASJ, and inner labials- IL1 and IL2 are known to fill with DiI (Burket *et al.*, 2006; Shaham, 2006; Starich *et al.*, 1995; Tong and Burglin, 2010) (Fig. 1B, C). In *S. hermaphroditum*, we found that neurons identifiable by position as analogs of the amphid neurons ASI, ADL, ASK, ASJ and the inner labial neurons IL1, and IL2 could be labeled using DiI filling (Fig. 1D-G). While amphid neurons are generally labeled in DiI dye-filling assays in *C. elegans* (Tong and Burglin, 2010), they were only intermittently stained in *S. hermaphroditum* (at least 1 putative amphid neuron is stained in roughly 20% of the treated animals). On the other hand, the inner labial neurons are much more accessible (approximately 90% of the treated animals have at least 2 putative IL neurons stained), including the IL1 neuron that is not commonly filled in *C. elegans* (IL1 staining in *C. elegans* is possible as shown in https://www.wormatlas.org/). Our observations are consistent with what has been observed in *S. carpocapsae*, in which putative IL2 neuron is dye accessible, and amphid neurons are stained only occasionally (Han *et al.*, 2015). While our observation focused on the head neurons, it is known that in the tail phasmid neurons, PHAs and PHBs can also be labeled using dye-filling in *C. elegans* (Hedgecock *et al.*, 1985; Tong and Burglin, 2010). However, we did not look for phasmid neurons in *S. hermaphroditum* hermaphrodites and it is unclear whether they were stained. As in *S. carpocapsae* (Han *et al.*, 2015), no dye-filling was observed in the stress-resistant IJ animals (Fig. 1J). By contrast, staining is observed in *C. elegans* dauers (Peckol *et al.*, 2001). The mechanism of dye-filling is not well understood, but the difference among species in uptake is likely caused by structural divergences in the cilia opening. Similarly, *Heterorhabditis bacteriophora,* another EPN, despite that it is evolutionarily much closer to *C. elegans* than that of *S. hermaphroditum*, is reported to have a similar staining pattern: their putative amphid neurons are not stained consistently, and their IJs are not stained (Han *et al.*, 2015). Besides dye filling in the head neurons of non-IJ animals, we also observed dye uptake by the excretory pore on the ventral surface of the animal near the head and in the gut of nearly every worm observed (Fig. 1H, I). Overall, we showed that dye-filling assays could be applied in *S. hermaphroditum*, and theanatomic characterization based on the staining suggests that it has a neuroanatomy similar to that of other characterized nematodes.

## Methods

**Nematode maintenance**

The handling of *C. elegans* and *S. hermaphroditum* was done as described in Brenner (1974) and Cao *et al.* (2021), respectively. Briefly, the wild‑type *C. elegans* strain N2 (Bristol) was cultured on Nematode Growth Medium (NGM) agar in 6 cm Petri dishes with a lawn of *E. coli* strain OP50 at 20°C; the wild‑type *S. hermaphroditum* strain PS9179 was cultured on NGM agar in 10 cm Petri dishes with a lawn of *Xenorhabdus griffiniae* strain HGB2511 at 25°C. OP50 and HGB2511 were cultured at 37°C and 30°C, respectively, before seeding lawns on NGM agar. Infective juveniles were obtained by water trap as previously described (Cao *et al.*, 2021).

**Dye filling assay**

Dye filling assay was adapted from a previously described method (Tong and Burglin, 2010) with modifications as described in the main text and as described below. Briefly, well-fed *S. hermaphroditum* was washed three times with 0.5% Triton X-100 in M9 and incubated on a shaker with DiI solution with a final concentration of 20µg/ml in M9 containing 0.5% Triton X-100 by volume, unless indicated otherwise. The DiI (1,1′-Dioctadecyl-3,3,3′,3′-Tetramethylindocarbocyanine Perchlorate, Invitrogen D282) was dissolved in dimethylformamide and stored in -20°C as a stock solution at 20 mg/ml. After 5 hours at 25°C, worms were again washed three times with 0.5% Triton X-100 in M9. Worms were then mounted on a 5% agarose pad with a drop of M9 buffer containing 20 mM sodium azide for observation.

**Dye filling assay optimization**

The staining protocol was optimized in 3 stages. First, we tested the effectiveness of the staining in 3 different buffers (0.5% Triton X-100 in M9, H2O, M9) at 10µg/ml dye concentration and an incubation time of 10 hours. The staining was best with 0.5% Triton X-100 in M9 and thus was used in subsequent experiments. We then tested the dye concentration using the same condition but with the same buffer (0.5% Triton X-100 in M9), in which we found 20 µg/ml to be the most suitable. Finally, we tested the staining duration (15 hours, 10 hours, 5 hours, 2 hours) using the condition mentioned above (0.5% Triton X-100 in M9 and 20 µg/ml dye) and concluded that a 5-10 hr incubation time would be optimal. The experiment presented in figure 1A was done later with the optimized condition except for one of the parameters.

**Image acquisition**

Photomicrographs were acquired with a Zeiss Imager Z2 microscope equipped with Apotome 2 and Axiocam 506 mono using Zen 2 Blue software.
